# Thermal photonics boosts radiative cooling

**DOI:** 10.1038/s41377-021-00691-7

**Published:** 2022-02-11

**Authors:** Ding Zhao, Min Qiu

**Affiliations:** 1 Key Laboratory of 3D Micro/Nano Fabrication and Characterization of Zhejiang Province, School of Engineering, Westlake University, 18 Shilongshan Road, Hangzhou, 310024 Zhejiang Province China; 2 Institute of Advanced Technology, Westlake Institute for Advanced Study, 18 Shilongshan Road, Hangzhou, 310024 Zhejiang Province China

**Keywords:** Mid-infrared photonics, Micro-optics

*Nature Photonics* (2022)

10.1038/s41566-021-00921-9

Thermal radiation is commonplace in our everyday life, exemplified by natural sunlight and infrared thermometers. When an object emits thermal radiation, a radiative cooling process carrying away energy from the object occurs spontaneously. Hence, the control of thermal radiation or radiative cooling is beneficial not only to the development of practical cooling techniques, but also to the exploitation of renewable energy resources. An emerging field of thermal photonics provides exciting opportunities for manipulating the radiative process artificially. In this review article, Shanhui Fan from Stanford University and Wei Li from Changchun Institute of Optics, Fine Mechanics and Physics, Chinese Academy of Sciences have discussed fundamental concepts involved in radiative cooling and summarized principles for tailoring thermal radiation with photonic structures. The story starts with the demand of daytime radiative cooling and introduces photonic concepts and recent advances in this area. Inspired by the daytime radiative cooling, more scenarios such as solar cell cooling, thermal management of outdoor colored objects and cooling textiles have been proposed. Thermodynamics in radiative cooling is finally discussed for harvesting outgoing thermal radiation. We anticipate these fruitful discussions can help readers walk into thermal photonics and motivate researchers to find novel applications of radiative cooling.

